# A prospective study of acute sinusitis, clinical features and modalities of management in adults, Sudan

**DOI:** 10.1186/s43163-022-00316-9

**Published:** 2022-10-03

**Authors:** Osama M. Khalid, Mashair B. Omer, Siddig E. Kardman, Hashim I. Yagi

**Affiliations:** 1 Department of Otolaryngology, Faculty of Medicine, University of Khartoum, Khartoum, Sudan; 2 Khartoum ENT Teaching Hospital, Khartoum, Sudan

**Keywords:** Acute sinusitis, Management, COVID-19, Adults, Sudan

## Abstract

**Background:**

Acute sinusitis is not an uncommon disease that manifests with inflammation of the mucosal lining of the paranasal sinuses. It has varied etiologies including viral, bacterial, fungal, and allergic. Anatomical variations, trauma, auto-immunity, diabetes mellitus, and dental procedures are predisposing factors. With the wide variation in the etiological factors, the management could be tricky. This study is quite relevant with the advent of the relentlessly persisting COVID-19 pandemic which affects the upper respiratory tract as well.

**Methods:**

This is a descriptive hospital-based prospective study conducted at the Khartoum ENT Teaching Hospital, Ibnsina Teaching Hospital, Omdurman Military Hospital, and Omdurman Teaching Hospital in Khartoum State in the period from March 2020 to February 2021. The study included all patients 18 years and older diagnosed with acute sinusitis. The data was collected by a well-structured questionnaire designed to meet the objectives of the study and analyzed using SPSS 20. Any COVID-19 suspect is excluded from the study.

**Results:**

The total number of patients was 109; of them, 59 (54.1%) were females and 50 (45.9%) were males, and the female to male ratio was 1.18:1. One hundred seven (98.2%) patients received medical treatment and two patients (1.8%) did take the medications. Eighty-one patients (74.3%) were cured with medical treatment and only 28 patients (25.7%) needed surgical intervention. The age group from 25 to 40 years old was the most affected, accounting for 68 patients (62.4%), and the above 60 years old (3.7%) was the least affected group.

**Conclusion:**

Acute sinusitis is not an uncommon disease, if addressed properly and timely is medically treatable in most cases apart from complicated cases. This study shows that the active working ages (25-40) were the most affected. Few patients needed surgery (FESS). Negligence could result in complications. Diseases like COVID-19 affect the upper respiratory tract, and there is a similarity in symptoms, and in the advent of the COVID-19 pandemic nowadays, differentiation is of paramount importance.

## Background

Rhinosinusitis is a symptomatic inflammation of nasal mucosa and paranasal sinuses. The term rhinosinusitis is preferable to sinusitis since inflammation of the sinuses rarely occurs without concurrent inflammation of the nasal mucosa. Acute sinusitis is defined as 2–4 weeks of purulent nasal discharge accompanied by nasal obstruction, facial pain, and facial pressure or fullness [1]. The mucosal mucus-secreting goblet cell and the cilia are physiologically important. Their damage leads to the accumulation of fluid predisposing to infection. The typing and etiology of acute sinusitis follow the causative factors. The viral sinusitis lasting for less than 10 days and not worsening, rhinovirus, adenovirus, influenza virus, and parainfluenza virus are the commonest causes. The acute bacterial sinusitis last more than 10 days, commonly caused by *Streptococcus pneumoniae*, *Hemophilus influenza*, and *Moraxella catarrhalis*. The acute fungal though rare does occur and is more likely to occur in immunocompromised patients, typical causing species include mucor, rhizopus, reconductor, and aspergillus. Aero-sinusitis occurs after flights [2]. Predisposing factors include diabetes mellitus, swimming, diving, high altitude, climbing, dental infection and dental procedures, trauma, and barotraumas [3]. As a clinical picture, general symptoms include fever, headaches, anosmia, and aural fullness, and the local cardinal features are nasal discharge, nasal obstruction, facial pain, and facial pressure [4]. The signs include tenderness to pressure and percussion of the sinuses, pain to pressure on the forehead, purulent secretions in the nasal cavity, post nasal discharge, pain behind the eye to pressure on the eye, pain to pressure on the cheek, and occasionally proptosis.

As for COVID-19, though they share similar symptoms, COVID-19 tends to have more general symptoms (shortness of breath or difficulty in breathing, chest pain, nausea, vomiting, diarrhea), and although, in both, you may lose your sense of taste and smell, but in COVID-19, losing your sense of taste and smell can happen without nasal congestion. Nonetheless, COVID-19 is unpredictable and could occur in asymptomatic carriers; others all of a sudden require mechanical ventilation progress to sepsis and organ failure. The management of acute sinusitis can be categorized into medical and surgical. The medical treatment consists of rest in bed, rehydration, analgesics, antipyretics, nasal decongestants, mucolytics, occasionally steroids, and lastly antibiotics. The surgical treatment is for those who fail medical treatment or develop complications, and it includes mainly functional endoscopic sinus surgery [5].

## Methods

This is a descriptive hospital-based study conducted at Khartoum ENT Teaching Hospital, Ibnsina Teaching Hospital, Omdurman Military Hospital, and Omdurman Teaching Hospital in Khartoum State in the period from March 2020 to February 2021. The study included all patients 18 years and older presenting to the above hospitals diagnosed with acute sinusitis. The data is collected by a well-constructed and designed questionnaire to achieve the objectives of the study and analyzed using SPSS20. Any COVID-19 suspect is excluded from the study.

## Results

This is a descriptive study in which a total number of 109 patients was included to study the management of acute sinusitis in adults. Regarding the age of the patient, the age group 25–40 years old showed the highest incidence, accounting for 68 patients (62.4%), and the group 40–60 was next accounting for17.4% followed by the group 10–25 16.5% and least was the group over 60 years 3.7%.

As for the gender of patients, females were 59 (54.1%) and males were 50 (45.9%), female to male ratio 1.18:1. Regarding the geographical distribution of patients, 66 were from the Khartoum state (60.6%) and 20 from the Jazeera state (18.3%) followed by the Darfur state 15 (13.8%), Kordofan state 7 (6.4%), and Gadarif 1 (0.9%).

**Fig. 1:**
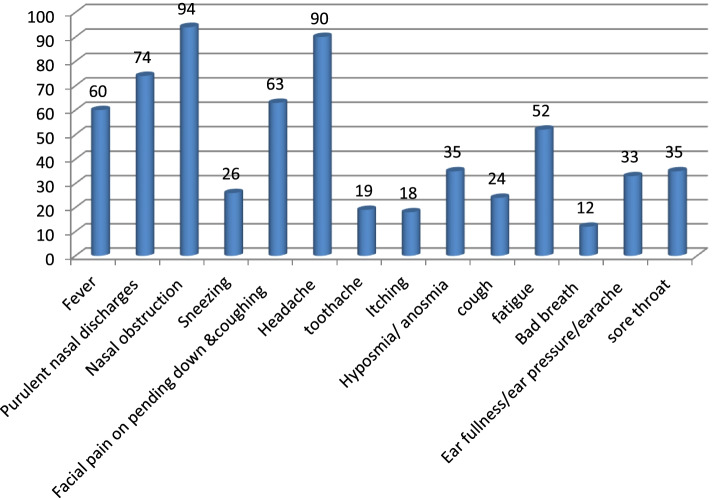
Representation of symptoms of patients with acute sinusitis

Regarding the symptoms of the disease, the most found symptom was nasal obstruction in 86.2% followed by purulent nasal discharge in 67.9%, facial pain on bending 57.8%, fever 55.0%, fatigue 47.7%, anosmia 32.1%, sore throat 32.1%, ear fullness 30.3%, sneezing 23.9%, cough 22.0%, toothache 17.4%, itching 16.5%, and halitosis 11.0% (Fig. 1).

Regarding the duration of symptoms, the minimum was 1 day and the maximum duration was 14 days, with a mean duration of 5.62. Most patients (*n*=87) were diagnosed in 1–7 days (79.8%) followed by 22 patients (20.2%) diagnosed in 8–14 days.

**Fig. 2:**
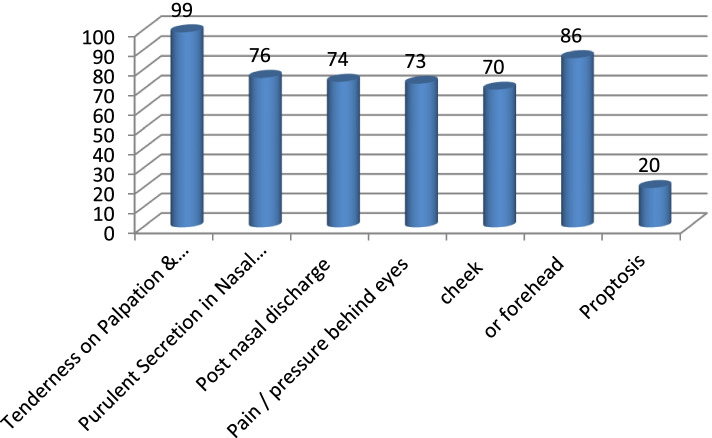
Distribution of signs of patients with acute sinusitis

As for signs of patients, the most common was tenderness to palpation and percussion of the sinuses elicited in 99 patients (90.8%) followed by pain to pressure on the forehead in 86 patients (78.9%), purulent secretions in the nasal cavity in 76 patients (69.7%), postnasal discharge in 74 patients (67.9%), pain behind the eye to pressure on the eye in73 patients (67.0%), pain to pressure on the cheeks in 70 patients (64.2%), and proptosis in 20 patients (18.3%). See Fig. 2.

Regarding the investigation, a CT scan of PNS was done for 53 (48.6), 47 patients (43.1%) were not investigated, and 9 patients (8.3%) did PNS x-ray.

Regarding the medical treatment used, nasal decongestants were prescribed for 86.2% followed by antibiotics for 76.1%, corticosteroid spray for 73.4%, antihistamine for 67.9%, analgesic for 60.6%, saline solution-douching spra*y* for 33.9%, oral corticosteroid for 16.5%, hydration for 15.6%, and humidification for 5.5%, while 1.8% of patients did not receive medical treatment.

Regarding side effects of treatment, most patients had no side effects 91.7%, only 8.3% reported side effects.

**Fig. 3:**
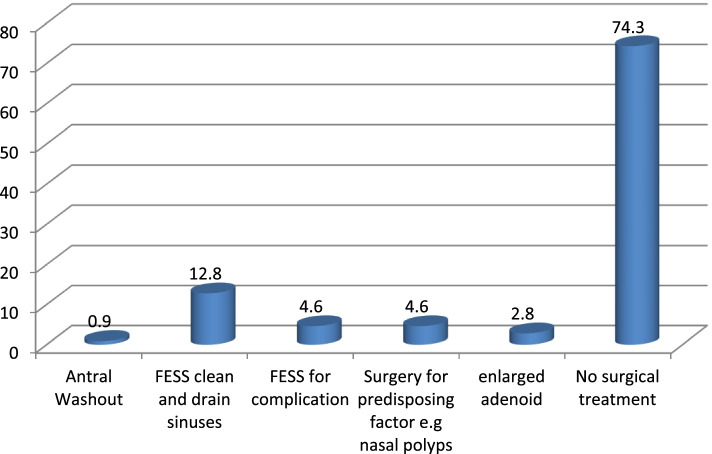
Distribution of surgical treatment of patients with acute sinusitis

As for the patients who needed surgery, FESS cleaning and draining of the sinuses was adopted in 12.8% of the cases (*n*=14), FESS for management of complications in 4.6% of the cases (*n*=5), surgery for predisposing factors (nasal polyps) in 4.6% of the cases (*n*=5), surgery of predisposing factors such as enlarge adenoid in 2.8% of the cases (*n*=3), and antral washout in 0.9% of the cases (*n*=1) (Fig. 3).

Regarding the outcome of treatment, most of the patients were cured (89.9% (*n*=98)), and 10.1 of the cases still needed further revision.

## Discussion

This study is an analytical hospital-based cross-sectional descriptive study conducted at the Khartoum ENT Teaching Hospital, Ibnsina Hospital, Omdurman Military Hospital, and Omdurman Teaching Hospital in Khartoum State. One hundred nine patients were included to study the management of acute sinusitis in adults among patients in the Khartoum state. In this advent of COVID-19 which is relentlessly persisting, this study is timely beneficial because of the similarity in symptoms.

Regarding the age of patients, most were in the age group 25–40 years old, 62.4%. This is an important age in which work morbidity is not affordable. The age group from 40–60 years old 17.4% followed, then 10–25 years old 16.5%, and the age group above 60 years 3.7% was the least affected. Probably due to recumbency they were less exposed. Yagi [6] reported that the most affected age was 21–30 years. Although our figures are not the same we are almost there when it comes to the age groups [6]. As for the gender distribution females were 59 (54.1%) and males were 50 (45.9%). Abdul et al. [7] reported sex distribution of patients diagnosed with sinusitis showed that out of 128 patients, 65 (51%) patients were males and 63 (49%) were females. Yagi [6] reported in his study that males were more than females. Our study agrees with ABDL K. Regarding the residential distribution of patients, most patients were from the Khartoum state 60.6% and Jazeera 18.3% followed by Darfur 13.8%, Kordofan 6.4%, and Gadarif 0.9%; the low figures of other states are not representative because patients from other states are just guest patients in Khartoum. Most patients complained of nasal obstruction 86.2% followed by headache 82.6% and purulent nasal discharge 67.9%, facial pain on pending 57.8%, fever 55.0%, fatigue 47.7%, anosmia 32.1%, sore throat 32.1%, ear fullness 30.3%, sneezing 23.9%, cough 22.0%, toothache 17.4%, itching16.5%, and halitosis 11.0%. Most patients have tenderness on palpation and percussion of the sinuses 90.8% followed by pain on pressure on the forehead 78.9%, purulent secretions in the nasal cavity 69.7%, postnasal discharge 67.9%, retroorbital pain on pressure on eyes 67.0%, pain on pressure on cheeks 64.2%, and proptosis 18.3%. Williams et al. reported the following symptoms were most sensitive; in his study, in adults, the presence of colored discharge, cough, and sneezing [8]. The symptoms and signs show similarity to a great degree in different studies though the order of frequency may vary.

We found the minimum duration of symptoms was 1 day and the maximum duration of symptoms was 14 days with a mean duration of symptoms of 5.62. Most patients (*n*=87) were diagnosed in 1–7 days 79.8%, and 20.2% were diagnosed in 8–14 days. Those who did CT scan PNS were 48.6% of the patients, among whom were the patients who failed medical treatment or developed complications; 8.3% did PNS X-Ray and 43.1% of the patients had no radiological investigations. Roxanne et al. [8] reported the diagnosis of sinusitis is usually made on clinical grounds. Apparently, the radiology is essential only in a selection of cases.

Regarding medical treatment, the most used was nasal decongestant in 86.2% followed by 76.1% of patients who received antibiotics, corticosteroid spray 73.4% antihistamine 67.9%, analgesic 60.6%, saline solution-douching spray 33.9%, oral corticosteroid 16.5%, hydration 15.6%, and humidification 5.5%. 1.8% of patients did not receive medical treatment. Rosenfeld et al. [9] reported in acute viral sinus infection pain relievers, nasal steroid sprays, and/or nasal saline rinses may be recommended. Antibiotics are not used for a viral sinus infection. Modalities of treatment and the medications used are variable but basically the same. In our study antibiotics were used in selected cases with a suspected bacterial infection. Adjuvants may be added. Rosenfeld et al. [9] reported if a decision is made to treat acute bacterial sinus infection with an antibiotic, amoxicillin or amoxicillin with clavulanate is the likely choice to be prescribed.

The rationale of treatment though mentioned in different words is basically the same.

Regarding surgical treatment, 74.3% of cases (*n*=81) did not need surgery. Patients treated by FESS cleaning and drainage of sinuses were adopted in 12.8% of the cases (*n*=14), FESS for complication in 4.6% of the cases (*n*=5), surgery for predisposing factor (nasal polyps) in 4.6% of the cases (*n*=5), surgery for predisposing factors such as enlarge adenoid in 2.8% of the cases (*n*=3), and antral washout 0.9% of the cases (*n*=1).

Yagi H reported in his study that the majority of patients were cured by medical treatment [6]. Surda P et al. (2016) reported the majority of patients with sinusitis do not require surgery [10–15].

The findings of our study go in consensus with the above studies [16–23]. The overall outcome of treatment showed most of the patients were cured 89.9% (*n*=98) and 10.1% of cases were still morbid and needed further intervention [24–27]. What makes our study unique and timely informative and different from previous studies in the advent of the relentlessly persisting COVID-19, it alerted to the similarity in symptoms. No case of COVID-19 was detected among the acute sinusitis patients in this study.

## Conclusion

Acute sinusitis is not an uncommon disease, if addressed properly and timely is medically treatable in most cases apart from complicated cases. Our study showed that the active working age group 25–40 is affected more. Negligence could result in complications. In this advent of COVID-19, both diseases affecting the upper respiratory with similarity in symptoms, differentiation is of paramount importance.

## Data Availability

All relevant data and methodological details pertaining to this study are available to interested researchers upon reasonable request to the corresponding author.

## Abbreviations

RS: Rhino sinusitis
ARS: Acute rhino sinusitis
CRS: Chronic rhino sinusitis
CT: Computed tomography
FESS: Functional endoscopic sinus surgery
GP: General practice
MRI: Magnetic resonance imaging
PNS: Para nasal sinuses
SPSS: Statistical package for the social sciences
URTI: Upper respiratory tract infection
MD: Diabetes mellitus
CSF: Cerebrospinal fluid
OTC: Over-the-counter
